# Supplementary material for: Disparities between native americans and white individuals in global outcome trajectories over the 5 years after traumatic brain injury: A model systems study
Source: PLoS One. 2025 Apr 8;20(4):e0321279. doi: 10.1371/journal.pone.0321279 (PMC11978045; doi:10.1371/journal.pone.0321279)
Supplement: Attachment — Submitted filename: Nuovo Documento di Microsoft Word.docx [file pone.0321279.s001.docx]

**Review Attachment**

Line 38: Would "ethnic group" be a better term? I understand that in the U.S. there is the concept of racial medicine, but since this manuscript will be read internationally, I suggest using a more inclusive term like "ethnic group."

Line 49: "Ethnic group."

Line 59: Please include the acronym for the CDC.

Line 104: "Ethnic group."

Line 107: "Ethnic minorities."

Lines 116-117: When hypothesizing higher rates of TBI within the Native American population, only papers from 2004 are cited. Could you please include more recent references?

Line 118: "Ethnic gender."

Line 119: "Ethnic group."

Line 120: Could you please add more citations to support your argument?

Line 121: "Ethnic group." Additionally, to further support your argument, could you please reference other sources beyond (35-38)? Perhaps more recent studies published in high-impact journals.

Line 124: A citation is missing. Since we are still in the introduction, personal opinions or hypotheses should not be presented at this point in the manuscript.

Line 150: "Ethnic health."

Line 158: Please avoid referring to figures present in the manuscript within the introduction section.

Line 159: Why is this citation included? You are discussing a model that has been utilized in the current study.

Line 163-170: It might be helpful to present the hypotheses and the research objective in a less schematic and more fluid, linear manner.

Line 167: I would suggest removing Hypothesis 3 since it is not tested in this study.

Lines 199-202: This section may be better suited for the Results rather than the Methods. The Methods should specify only the statistical tests used, without previewing the results.

Line 200: Before performing the ANOVA test, were the data checked for normal distribution?

Line 212: "Ethnicity."

Lines 213-216: I believe this line may not be necessary for the manuscript.

Line 173: Consider a more descriptive title for this paragraph.

Lines 217-218: Please consider a more unique and comprehensive title for this paragraph.

Lines 238-248: This explanation might be more appropriate in the Introduction rather than the Methods section, as the Methods should focus on what was done specifically in this study.

Line 246: Incorrect citation. The number should be provided instead of "Wilson et al., 1998."

Line 251: The unit is missing.

Line 253: "Ethnicity."

Line 272: "Ethnicity."

Line 281: The unit is missing.

Line 296: "Ethnicity."

Line 300: p = 0.009 (?); "Ethnicity."

Line 308: "Ethnicity."

Line 309: p = 0.348 (?)

Line 310: p < 0.01 (?)

Line 312: "Ethnicity."

Line 316: "Ethnicity."

Line 318: "Ethnicity"; p = 0.139 (?)

Line 319: p < 0.001 (?)

Table 1: In the entire p-value column, the units are missing. It might also be helpful to highlight the significant p-values more clearly. Additionally, a reference to the type of test conducted should be included.

Line 690: If I understand correctly, this is the model used by Meade et al., 2015. I suggest specifying in the figure description that this model is a reworking of a previous model.

Figure 2: Please adjust the x-axis by setting the numbers at regular intervals (e.g., 1, 2, 3, 4, 5) and labeling the time (years).
