# Supplementary material for: Disparities between native americans and white individuals in global outcome trajectories over the 5 years after traumatic brain injury: A model systems study
Source: PLoS One. 2025 Apr 8;20(4):e0321279. doi: 10.1371/journal.pone.0321279 (PMC11978045; doi:10.1371/journal.pone.0321279)
Supplement: Attachment — Submitted filename: Comments to Reviewers 10-09-24.docx [file pone.0321279.s002.docx]

Dear Dr. Perrin,

Thank you for submitting your manuscript to PLOS ONE. After careful consideration, we feel that it has merit but does not fully meet PLOS ONE’s publication criteria as it currently stands. Therefore, we invite you to submit a revised version of the manuscript that addresses the points raised during the review process.

Comments from the editorial office: Upon internal evaluation of the reviews provided, we kindly request you to disregard the reviewer report provided by Reviewer 2. No amendments are required in response to Reviewer 2’s comments. ’

Please submit your revised manuscript by Oct 19 2024 11:59PM. If you will need more time than this to complete your revisions, please reply to this message or contact the journal office at [plosone@plos.org](mailto:plosone@plos.org). When you're ready to submit your revision, log on to <https://www.editorialmanager.com/pone/> and select the 'Submissions Needing Revision' folder to locate your manuscript file.

Please include the following items when submitting your revised manuscript:

- A rebuttal letter that responds to each point raised by the academic editor and reviewer(s). You should upload this letter as a separate file labeled 'Response to Reviewers'.
- A marked-up copy of your manuscript that highlights changes made to the original version. You should upload this as a separate file labeled 'Revised Manuscript with Track Changes'.
- An unmarked version of your revised paper without tracked changes. You should upload this as a separate file labeled 'Manuscript'.

If you would like to make changes to your financial disclosure, please include your updated statement in your cover letter. Guidelines for resubmitting your figure files are available below the reviewer comments at the end of this letter.

If applicable, we recommend that you deposit your laboratory protocols in [protocols.io](http://protocols.io/) to enhance the reproducibility of your results. Protocols.io assigns your protocol its own identifier (DOI) so that it can be cited independently in the future. For instructions see: [https://journals.plos.org/plosone/s/submission-guidelines#loc-laboratory-protocols](https://c05y1x9s.r.us-east-2.awstrack.me/L0/https:%2F%2Fjournals.plos.org%2Fplosone%2Fs%2Fsubmission-guidelines%23loc-laboratory-protocols/1/010f0191c0aef239-fa23fe07-7c91-4ca9-9937-2050ca29611d-000000/DU9nlc3Ie_0X05k5s7qFa5e62DU=175). Additionally, PLOS ONE offers an option for publishing peer-reviewed Lab Protocol articles, which describe protocols hosted on [protocols.io](http://protocols.io/). Read more information on sharing protocols at [https://plos.org/protocols?utm_medium=editorial-email&utm_source=authorletters&utm_campaign=protocols](https://c05y1x9s.r.us-east-2.awstrack.me/L0/https:%2F%2Fplos.org%2Fprotocols%3Futm_medium=editorial-email%26utm_source=authorletters%26utm_campaign=protocols/1/010f0191c0aef239-fa23fe07-7c91-4ca9-9937-2050ca29611d-000000/i7FLidg-tdMNcPiLzGj_cZPHlD0=175).

We look forward to receiving your revised manuscript.

Kind regards,

Annesha Sil, Ph.D.
Associate Editor
PLOS ONE

**Reviewer #1:**

This paper was easy to read, used appropriate methods to assess the hypotheses, and addressed limitations as well as addressed future directions for research and clinical considerations for health care professionals. As such, this paper should assist clinicians and researchers in this area for future research and improvement in healthcare delivery for the relevant populations.

*Thank you so much for your kind words and positive review of our manuscript. We are thrilled that you think it is a meaningful contribution to the literature!*

**Reviewer #2:**

Comments from the editorial office: Upon internal evaluation of the reviews provided, we kindly request you to disregard the reviewer report provided by Reviewer 2. No amendments are required in response to Reviewer 2’s comments.

*We have followed the editorial office’s guidance to disregard this reviewer report.*

**Reviewer #3:**

First of all, thanks a lot to the authors' research team for their efforts in this manuscript. I think this manuscript is innovative and extraordinarily clinically relevant. However, there are still some small problems with this manuscript that need further improvement:

*Thank you so much for your positive comments especially regarding the innovation and clinical relevance!*

1. In the Introduction section, the author describes the epidemiology, causes, and negative effects of traumatic brain injury in Native Americans and White Individuals. The “data differences” between the two in terms of prevalence, causes, and negative effects are also noted. However, this can still only be defined as a detailed introduction to the background, without clearly identifying the clinical significance of the study. I would suggest that the authors streamline some of the content in the introduction section and add a few points of necessity for the purpose of this study in the context of this background.

*Thank you for this comment. We have now added the following language to the Study Purpose section, highlighting key gaps in the literature and how our study helps fill these gaps: “Given the extensive differences in the causes of TBI, comorbidities, and outcomes between Native Americans and White individuals, the current study utilized a modified version of the MHDD (Figure 1) which has been previously used to examine disparities between Native Americans and White individuals with TBI (32) to investigate the degree to which disparities in global outcomes exist between the two groups over the 5 years post-injury. Further, only one other study to date has investigated how demographic and TBI injury characteristics differ between Native Americans and White individuals in a demographically matched sample (32). The current study highlighted demographic and injury-related characteristics that differed between the two groups then used these statistically significant differences to attempt to account for the disparity in global outcomes.”*

2. What the authors gained in the Results section was very fruitful, but they failed to have a full one-on-one discussion in the Discussion section.

*The primary results for the current study were: (1) Native Americans were less likely to be employed at the time of injury than White individuals, (2) Native Americans were less likely to have private insurance at the time of injury than White individuals, (3) Native Americans were more likely to report having used illicit/non-prescription drugs in the month prior to injury than White individuals, (4) Native Americans were more likely to report binge drinking in the month prior to injury than White individuals, (5) Native Americans reported worse global outcome scores both generally and over time, and (6) the difference in global outcome scores, both generally and over time, was accounted for by the addition of the sociodemographic characteristics that differed between the two groups.*

*The first paragraph of the Sociodemographic Differences section now discusses the results that Native Americans were less likely to be employed at the time of injury than White individuals. We have now added lines regarding private health insurance to greater emphasize these findings and offer a theory about how it might impact TBI rehabilitation.*

*The second paragraph under the Sociodemographic Differences section discusses substance use as a whole (i.e., both illicit non-prescription drug use and alcohol use). We have now reworked this paragraph to ensure this is more clearly stated. The differences in global outcome are now discussed in detail over the next two pages. We hope these changes help create a better one-on-one discussion of the results.*

3. Although the author makes many thought-provoking points in Global Outcome, it spans a wide range from the clinical to the societal level, so would it be possible to integrate them again in a separate paragraph.

*In the hopes of improving readability and integrating the results section into one, cohesive paragraph, the following paragraph was added to the end of the Discussion section: “The MHDD theorizes that health-related outcomes for individuals with disability are the result of interactions between a wide variety of factors. The current study reinforces this model by providing a picture of disparities in global outcome between Native Americans and White individuals with TBI that is complex and intersectional. Employment probabilities, type of insurance, illicit/non-prescription drugs, alcohol use, and systemic barriers may all play a role in predicting global outcome scores and disparities for Native Americans with TBI. Type of insurance appears to be particularly important as it was the only predictor to remain significant across all models.”*

4. In addition, perhaps the author should also pay attention to standardize some writing details, for example, “TBI” in the Introduction section should be expressed as Traumatic brain injury (TBI). Abbreviations in the Abstract should not be used directly in the text. Abbreviations that appear for the first time in the text should be labeled as such. (line 57)

*We have scanned the document and found two abbreviations used in the abstract that were not also expressed in the text of the manuscript. TBI has now been expressed as traumatic brain injury in the opening paragraph of the introduction, and TBIMS has been expressed as the Traumatic Brain Injury Model Systems in the first paragraph of the Procedure section.*

There is also a problem with the formulation of this sentence: “Falls, collisions with an object, and motor vehicle accidents are leading causes of TBI in the U.S., while suicide, unintentional falls, and motor vehicle accidents are leading causes of TBI in the U.S.,” and the statement “TBI in the U.S. is the most common cause of TBI. unintentional falls, and motor vehicle accidents are the leading causes of TBI-related deaths in the U.S (1,4).”

*We have now changed both of these to: “Falls, collisions with an object, and motor vehicle accidents are leading causes of TBI in the U.S., while suicide, unintentional falls, and motor vehicle accidents are the leading causes of TBI-related deaths in the U.S.”*

There are other similar issues with writing conventions in the text, and it is hoped that the authors' reworking will make this manuscript a greater improvement in the presentation of its content.

*We have conducted a thorough review of the document for other potential deviations from writing convention and corrected them wherever found.*

**Reviewer #4:**

I would like to thank the authors for submitting this manuscript. I believe the study has potential, especially given the lack of research on TBI and Native Americans.

*Thank you for reading our manuscript and for your comments recognizing the significant gap in the literature our research addresses.*

However, I think the research question needs to be better formulated, as it currently relies on hypotheses that are not well-supported scientifically. I recommend refining the hypotheses and research questions and supporting them with a more thorough literature review.

*We have changed the Hypotheses section to a newly title Study Rationale and Hypotheses section. We have added the following sentences to help integrate the information provided in the background to more cohesively justify our hypotheses. Additionally, while no information currently exists on global outcome trajectories, we have added four additional citations from the last 2 years justifying our approach through literature on other marginalized populations within the U.S. This section reads: “While no research to date has investigated global outcome for Native Americans, previously literature has shown that Native Americans face a plethora of barriers to recovery including: higher rates of depression and anxiety, lower healthcare utilization and access, and lower functional independence following TBI (28,32,42,43), indicating Native Americans are at a greater risk for negative complications following TBI when compared to White individuals. Further, while little research to date has investigated possible predictors of outcome following TBI for Native Americans, the literature for outcomes following TBI for other underserved populations (e.g., Hispanics) is growing, with evidence that sociodemographic factors like employment, alcohol use, marital status, violence as a cause of injury (47–50). Based on this literature, we hypothesize that…”*

Additionally, the sample size used in this study (75 Native Americans + 75 White individuals) seems too small to draw the conclusions presented in the manuscript, which is a significant limitation of the study.

*To address this concern, we have done several things. (1) We have added the following sentence to the Procedure and Study Approval section: “At present, no other database offers such nuanced data on Native Americans with TBI.” This sentence is intended to highlight the incredible uniqueness of the sample to study a construct that has never been investigated for Native Americans. (2) We have gone through the discussion section to attenuate the language surrounding the results, ensuring that the language does not imply causality. We have also extended the discussion section based on feedback from Reviewer 3 to include more detailed analysis of the results. (3) We have extended the limitation section to note (a) the uniqueness of the sample and (b) further expand the limitations on generalizability.*

Lastly, in the introduction, I suggest rephrasing the paragraph discussing TBI and Native Americans, as the current wording in the manuscript may come across as insensitive to this group. I recommend rewording the paragraph in a more considerate manner, using as inclusive language as possible.

*We have reviewed the section on TBI in Native Americans and have changed the language to reflect a more “ethnic” rather than “racial” approach as your line-items suggest. Additionally, we have added a few more citations to give the background literature greater context and assist with interpretation.*

Further comments can be found in the attached file.

Line 38: Would "ethnic group" be a better term? I understand that in the U.S. there is the concept of racial medicine, but since this manuscript will be read internationally, I suggest using a more inclusive term like "ethnic group."

*We have changed this to “ethnic group.”*

Line 49: "Ethnic group."

*We have changed this to ethnic group.*

Line 59: Please include the acronym for the CDC.

*We have no added “Centers for Disease Control and Prevention.”*

Line 104: "Ethnic group."

*We have changed this to “ethnic group.”*

Line 107: "Ethnic minorities."

*We have changed this to “ethnic minorities.”*

Lines 116-117: When hypothesizing higher rates of TBI within the Native American population, only papers from 2004 are cited. Could you please include more recent references?

*The references you have noted are specifically for higher rates of IPV related to TBI. In response to the reviewer’s question, we performed an additional very thorough search of the literature. Unfortunately, there are no more contemporaneous citations for this data. At the start of the “TBI in Native Americans” section, 6 citations are listed within the first two sentences that range in publication date from 2003-2023 in an attempt to showcase that the disproportionate rates of TBI have existed for at least the last 20 years or so.*

Line 118: "Ethnic gender."

*We have changed this to “ethnic gender.”*

Line 119: "Ethnic group."

*We have changed this to “ethnic group.”*

Line 120: Could you please add more citations to support your argument?

*We have now added an additional citation from the CDC that details violence as a cause of injury and an additional citation from 2016 that discusses IPV for Native Americans and how it may relate to increased risks of TBI.*

Line 121: "Ethnic group." Additionally, to further support your argument, could you please reference other sources beyond (35-38)? Perhaps more recent studies published in high-impact journals.

*We have changed this to “ethnic groups.” We have added a citation from 2020 discussing the possible link between alcohol use disorder and TBI. Unfortunately, substance use and TBI are grossly under-researched in Native Americans. The citations used in the manuscript are the most recent on this topic and are all from high impact journals within the last ten years.*

Line 124: A citation is missing. Since we are still in the introduction, personal opinions or hypotheses should not be presented at this point in the manuscript.

*We have now added a citation that discusses the links between alcohol use disorder, impaired driving, and TBI.*

Line 150: "Ethnic health."

*We have changed this to “ethnic health.”*

Line 158: Please avoid referring to figures present in the manuscript within the introduction section.

*We have now moved Figure 1 to the Study Measures - Demographic and Injury-Related Variables of the Method.*

Line 159: Why is this citation included? You are discussing a model that has been utilized in the current study.

*We have added the phrase “which has been previously” to the sentence to highlight that this model has a history of being used to investigate health disparities for Native Americans with TBI.*

Line 163-170: It might be helpful to present the hypotheses and the research objective in a less schematic and more fluid, linear manner.

*We have integrated the hypotheses into a paragraph and more fluid context.*

Line 167: I would suggest removing Hypothesis 3 since it is not tested in this study.

*We apologize for the confusion, but Hypothesis 3 is tested in the current study via the hierarchical linear multiple regressions run during the analyses. Primary Set 2 incorporates the significantly different sociodemographic variables between the two groups in an attempt to account for the variance in global outcome scores. The results demonstrate that Hypothesis 3 was rejected; that is, the difference in global outcomes scores did not persist after accounting for the significantly different sociodemographic variables between the two groups.*

Lines 199-202: This section may be better suited for the Results rather than the Methods. The Methods should specify only the statistical tests used, without previewing the results.

*We appreciate this commentary; however, this section deals directly with how we reached a sample size of 75 Native Americans and 75 White individuals with TBI. We believe this section fits most fluidly within the participants section as it is not part of the study results but how we formed our sample. If the reviewer or editor feels strongly that this should be moved, we are happy to do so.*

Line 200: Before performing the ANOVA test, were the data checked for normal distribution?

*We did not check the year of injury ANOVA for normal distribution, but upon a second reading, we actually do not think this exploratory analysis is important. We have now deleted it to streamline the manuscript better, as requested by the reviewers.*

Line 212: "Ethnicity."

*We have changed this to “ethnicity.”*

Lines 213-216: I believe this line may not be necessary for the manuscript.

*We have deleted this sentence.*

Line 173: Consider a more descriptive title for this paragraph.

*We have changed the title to “Procedure and Study Approval.”*

Lines 217-218: Please consider a more unique and comprehensive title for this paragraph.

*We have changed the title to “Study Measures.”*

Lines 238-248: This explanation might be more appropriate in the Introduction rather than the Methods section, as the Methods should focus on what was done specifically in this study.

*As this information already appears in the introduction, we have opted to delete it from the methods section.*

Line 246: Incorrect citation. The number should be provided instead of "Wilson et al., 1998."

*We have corrected this by appropriately formatting the citation. Apologies for missing it in the original submission.*

Line 251: The unit is missing.

*We have added the leading “0.”*

Line 253: "Ethnicity."

*We have changed this to “ethnicity.”*

Line 272: "Ethnicity."

*We have changed this to “ethnicity.”*

Line 281: The unit is missing.

*We have added the leading “0.”*

Line 296: "Ethnicity."

*We have changed this to “ethnicity.”*

Line 300: p = 0.009 (?); "Ethnicity."

*We have changed this to “ethnicity.” We have added the leading “0.”*

Line 308: "Ethnicity."

*We have changed this to “ethnicity.”*

Line 309: p = 0.348 (?)

*We have added the leading “0.”*

Line 310: p < 0.01 (?)

*We have added the leading “0.”*

Line 312: "Ethnicity."

*We have changed this to “ethnicity.”*

Line 316: "Ethnicity."

*We have changed this to “ethnicity.”*

Line 318: "Ethnicity"; p = 0.139 (?)

*We have made this change.*

Line 319: p < 0.001 (?)

*We have added the leading “0.”*

Table 1: In the entire p-value column, the units are missing. It might also be helpful to highlight the significant p-values more clearly. Additionally, a reference to the type of test conducted should be included.

*We have added the leading “0” for each number. We have also added a legend to help highlight significance at .05 and .001. Further, we have added “via HLM” to the title of the table to denote what type of test was conducted.*

Line 690: If I understand correctly, this is the model used by Meade et al., 2015. I suggest specifying in the figure description that this model is a reworking of a previous model.

*We have now added a note to the figure to specifically show that the study model is an adaptation of the MHDD.*

Figure 2: Please adjust the x-axis by setting the numbers at regular intervals (e.g., 1, 2, 3, 4, 5) and labeling the time (years).

*We have changed the figure to your requested specifications.*
