# Supplementary material for: Disparities between native americans and white individuals in global outcome trajectories over the 5 years after traumatic brain injury: A model systems study
Source: PLoS One. 2025 Apr 8;20(4):e0321279. doi: 10.1371/journal.pone.0321279 (PMC11978045; doi:10.1371/journal.pone.0321279)
Supplement: Attachment — Submitted filename: Response to Reviewers 2.docx [file pone.0321279.s003.docx]

**Comments to the Author**

1. If the authors have adequately addressed your comments raised in a previous round of review and you feel that this manuscript is now acceptable for publication, you may indicate that here to bypass the “Comments to the Author” section, enter your conflict of interest statement in the “Confidential to Editor” section, and submit your "Accept" recommendation.

Reviewer #1: All comments have been addressed

Reviewer #3: All comments have been addressed

Reviewer #5: (No Response)

2. Is the manuscript technically sound, and do the data support the conclusions?

The manuscript must describe a technically sound piece of scientific research with data that supports the conclusions. Experiments must have been conducted rigorously, with appropriate controls, replication, and sample sizes. The conclusions must be drawn appropriately based on the data presented.

Reviewer #1: Yes

Reviewer #3: Yes

Reviewer #5: Yes

3. Has the statistical analysis been performed appropriately and rigorously?

Reviewer #1: Yes

Reviewer #3: Yes

Reviewer #5: No

4. Have the authors made all data underlying the findings in their manuscript fully available?

The [PLOS Data policy](http://track.editorialmanager.com/CL0/http:%2F%2Fwww.plosone.org%2Fstatic%2Fpolicies.action%23sharing/1/010f019390479261-e23ca9a1-f283-4691-9bf0-8e7c3ab6e99c-000000/PXrizsNG8fzD4C7nBYZWi96r9J_SpWYKg1L9_s5YFEM=187) requires authors to make all data underlying the findings described in their manuscript fully available without restriction, with rare exception (please refer to the Data Availability Statement in the manuscript PDF file). The data should be provided as part of the manuscript or its supporting information, or deposited to a public repository. For example, in addition to summary statistics, the data points behind means, medians and variance measures should be available. If there are restrictions on publicly sharing data—e.g. participant privacy or use of data from a third party—those must be specified.

Reviewer #1: Yes

Reviewer #3: Yes

Reviewer #5: No

*We have now stated in the Procedure and Study Approval section that “The data are publicly available through an external data request to the TBIMS National Data and Statistical Center (https://www.tbindsc.org/researchers.aspx).”*

5. Is the manuscript presented in an intelligible fashion and written in standard English?

PLOS ONE does not copyedit accepted manuscripts, so the language in submitted articles must be clear, correct, and unambiguous. Any typographical or grammatical errors should be corrected at revision, so please note any specific errors here.

Reviewer #1: Yes

Reviewer #3: Yes

Reviewer #5: Yes

6. Review Comments to the Author

Please use the space provided to explain your answers to the questions above. You may also include additional comments for the author, including concerns about dual publication, research ethics, or publication ethics. (Please upload your review as an attachment if it exceeds 20,000 characters)

Reviewer #1: You have completed all revisions satisfactorily which in turn has made the paper even better. Excellent work.

Reviewer #3: (No Response)

Reviewer #5:

1. The revised introduction adds context to the study’s significance but fails to connect research gaps to actionable clinical or policy objectives. It focuses too heavily on statistical analysis while neglecting systemic barriers like access to culturally informed care or tribal healthcare limitations. Without addressing these issues, the introduction weakens the case for why these disparities matter for healthcare interventions and policy-making.

*We have now added information on a study that investigates disparities between Native Americans and White individuals for community participation following TBI. We have also added the following paragraph to the Introduction section to draw attention to general disparities/marginalization, healthcare specific disparities, and issues around cultural dissonance and provider mistrust faced by Native Americans:*

*“In addition to inequal outcomes following TBI, Native Americans are recognized as a severely underserved and marginalized population, facing disparities in diseases, healthcare utilization, poverty rates, and lower education and employment (46). Native Americans are significantly more likely to face premature mortality due in large part to disparities in chronic disease and lack of appropriate specialized healthcare providers in tribal lands or through the Indian Health Service (47). The lack of reliable and adequate healthcare services, heightened rates of high mortality-related injuries and diseases (e.g., TBI, diabetes), and suspicion of western medical practices and provider distrust, combine to create a bleak picture for the state of Native American healthcare (48,49).”*

2. Discussions of employment, insurance, and substance use findings were added, but they lack depth and fail to analyze systemic factors like geographic barriers or socioeconomic disadvantages. Private insurance is identified as a predictor, but its role within broader inequities is unexplored. The absence of unmeasured variables, such as healthcare trust or systemic racism, limits the analysis of disparities in long-term care for Native Americans.

*The first paragraph to the Sociodemographic Differences section was significantly updated and extended with several new citations and additional information as follows:*

*“In line with previous research suggesting Native Americans experience lower employment rates than the general U.S. population and within the TBI population (32,33,45), the current study found that Native Americans were significantly less likely to be employed prior to experiencing a TBI than their White counterparts. While information on employment and insurance rates for Native Americans is lacking, particularly for Native Americans with TBI, a separate TBIMS study utilizing a sample that partially overlaps with the present study also suggested Native Americans were less likely to be employed prior to injury and less likely to have private insurance than White individuals (32). These may be key contributing factors for why Native Americans are more likely to experience barriers to healthcare access and less likely to engage with the healthcare system as private insurance often affords greater and more varied access to care, and employment is often a primary means of acquiring health insurance (28,62). Private health insurance also typically affords greater and more varied access to healthcare than non-private alternatives. It is possible that Native Americans who lack private healthcare experience worse care or have a more difficult time accessing appropriate care, thus negatively impacting their recovery post-TBI. Given the higher rates of poverty within the Native American population coupled with their documented concerns about the high cost of healthcare, this may make Native Americans less likely to seek healthcare services even if they are available (48,63). Issues such as cultural dissonance, provider distrust, and suspicion of Western medical practices may further impede Native Americans’ decision to seek treatment (47,48). These concerns, coupled with higher rates of TBI and chronic comorbid conditions (e.g., diabetes) create a scenario in which Native Americans may need greater and more specialized healthcare but be less likely to engage (32,33,47,63).”*

*Further, we added additional information to the limitations section to highlight that the current study was unable to explore issues surrounding systemic barriers and highlighted specific variables that may be of interest for future studies: “Research indicates that other variables not assessed in the current study like systemic barriers (e.g., distance to the nearest hospital), quality of care provided, the number and severity of comorbid conditions, cultural dissonance between patient and provider may be important factors contributing to differential outcomes following TBI (32,33,45,47,48). Particularly given the limited research on racial experiences of Native Americans with healthcare systems and providers (70), future research may wish to investigate how racial bias may impact healthcare access and quality for Native Americans with TBI.”*

*This added section ties in directly with a section added to the Global Outcome section that directly addresses issues of systemic barriers/racism: “Finally, while not assessed in the present study, a plethora of research has indicated racism as a key factor contributing to healthcare inequalities (66–68). Especially within the U.S., some research has suggested that racism is embedded within institutions, legal systems, and social structures and continues to negatively impact racial/ethnic minority communities who are unfairly targeted or marginalized by the U.S. healthcare system (69). The limited literature on Native Americans with TBI indicates they experience high levels of racial discrimination (70) and are more likely to receive invasive surgery whereas White individuals are less likely and instead to be recommended for less invasive treatments like occupational or physical therapy, despite comparable TBI pathology (38). This, coupled with disparities in various outcomes (e.g., cognitive and motor function, depression) can paint a bleak picture for the state of care received by Native Americans and may be contributing to lower global outcome scores (32,43).*

3. The consolidation of the global outcomes discussion improves readability but does little to deepen the analysis. The authors fail to explain why disparities worsen over time or suggest interventions to address them. Specific components of the Glasgow Outcome Scale-Extended (e.g., family and social roles) that may disproportionately affect Native Americans remain unexamined, leaving a critical gap in understanding disparities.

*The following paragraphs have been expanded with additional information and citation in the hopes of filling the gaps mentioned above: “Type of insurance was also identified in the current study as a key source of disparities in global outcome scores between Native Americans and White individuals with TBI. This makes sense as private insurance can provide greater access to care and more varied resources. The lack of resource may result in less specialized care or force Native Americans to use rural healthcare centers or Indian Health Service facilities that may be ill-equipped to provide the type and quality of care necessary for more complex conditions like TBI (47). Given that Native Americans frequently experience barriers to healthcare services (e.g., cultural mistrust, racism) and are less likely to utilize healthcare services and resources and to receive or be recommended for more advanced services post-rehabilitation (28,38), it is possible that type of insurance may limit access for Native Americans and thereby reduce global outcome scores. While not assessed in the present study, it is possible the lack of private insurance and reduced healthcare service utilization may lead to the development of additional comorbid conditions (46).*

*Finally, while not assessed in the present study, a plethora of research has indicated racism as a key factor contributing to healthcare inequalities (66–68). Especially within the U.S., some research has suggested that racism is embedded within institutions, legal systems, and social structures and continues to negatively impact racial/ethnic minority communities who are unfairly targeted or marginalized by the U.S. healthcare system (69). The limited literature on Native Americans with TBI indicates they experience high levels of racial discrimination (70) and are more likely to receive invasive surgery whereas White individuals are less likely and instead to be recommended for less invasive treatments like occupational or physical therapy, despite comparable TBI pathology (38). This, coupled with disparities in various outcomes (e.g., cognitive and motor function, depression) can paint a bleak picture for the state of care received by Native Americans and may be contributing to lower global outcome scores (32,43).”*

*The following two sentences were added to the second paragraph of the Limitations and Future Directions section to, as the reviewer suggested, note the limitation of using a total GOS-E score rather than examine item-level differences: “The use of the total score for the GOS-E reduces some of the nuance that may be obtained by exploring item-level differences. Future studies may wish to use a larger sample to analyze differences in specific components of the GOS-E.”*

4. Revising the hypotheses and adding citations improves their context but fails to address concerns about causality. Hypothesis 3 remains untested, and it is unclear whether the added citations are specific to Native Americans or apply broadly to underserved populations. Explicitly acknowledging limitations in inferring causality would improve the framing of the hypotheses.

*We apologize for our lack of clarity. The third hypothesis is tested by “Primary Set 2” in which we run a second set of HLMs that include the statistically different covariates. To help with clarity on which hypotheses are tested by the various analysis steps, we have added notation for “hypothesis 1,” “hypothesis 2,” and “hypothesis 3” throughout the results section. We have also explicitly stated in the Primary Set 2 section “thus, for the study’s third hypothesis, we failed to reject the null.”*

*Based on feedback from previous reviewers we expanded the section in Limitations and Future Directions regarding the inability to infer causality. That section now reads as follows: “While the current study identified employment at the time of injury, substance use, and type of insurance as important factors accounting for global outcome disparities between Native Americans and White individuals, the exact mechanism for these relations was not uncovered in the current study. Despite the large number of sociodemographic and injury-related characteristics investigated, it is impossible to prove a causal relationship between these covariates and global outcome disparities. That is, the significantly different variables between the two groups could be markers for other unmeasured sources of variance that directly contribute to disparities in global outcome following TBI for Native Americans.”*

5. The authors justify the small sample size as reflective of the rarity of Native American TBI cases, but this does not address its impact on generalizability and statistical power. Power analyses are not discussed, and sensitivity analyses could help demonstrate the robustness of the findings despite this limitation. These omissions weaken the study’s credibility.

*To address the reviewer’s concerns regarding statistical power, we have added the following new information and analyses to the Results section:*

*“****Effect Sizes and Post-hoc Power Analysis.*** *Cohen’s d effect sizes were calculated for the differences between Native American and White Individuals’ GOS-E scores at years 1, 2, and 5 post-injury (Table 4). Year one was below the threshold of a small-sized effect; year two showed a small-sized effect, and year five showed a medium sized-effect. A post-hoc power analysis conducted a priori in G*Power 3.1.9.7 targeting a medium effect size with α = .05 and power at 0.80, revealed the need for a total sample size at N = 102, well below the 150 sample for the current study.*

| *Table 4* | | | |
| --- | --- | --- | --- |
| *Effect Sizes for GOSE at 1-, 2-, and 5-Years Post-injury by Race/Ethnicity* | | | |
| *Variable* |  | *Native Americans* | *White Individuals* |
|  | *Cohen's d* | *M (SD)* | *M (SD)* |
| *One Year GOSE* | *0.17* | *5.42 (1.84)* | *5.72 (1.68)* |
| *Two Year GOSE* | *0.20* | *5.42 (1.80)* | *5.78 (1.77)* |
| *Five Year GOSE* | *0.58* | *4.95 (1.98)* | *5.98 (1.65)* |

*Additionally, we have added the following details to the Limitations and Future Directions section in which we explicitly mention the difficulties surrounding generalizability as well as cite literature on the robustness of HLM and how the sample size of 150 individuals is more than adequate for the present analyses: “While the sample size of 150 Native American and White individuals is adequate for the present analyses, large enough to detect a medium effect size, (71,72) and unique within the rehabilitation literature to date, it makes generalizing to larger populations of Native Americans somewhat difficult given the culturally distinct and geographically isolated nature of Native American tribes within the U.S. (45). Further, the sample size is well below the sample size recommended by G*Power to detect a small sized effect (N = 620), further limiting the generalizability of the study’s results.”*

7. PLOS authors have the option to publish the peer review history of their article ([what does this mean?](https://track.editorialmanager.com/CL0/https:%2F%2Fjournals.plos.org%2Fplosone%2Fs%2Feditorial-and-peer-review-process%23loc-peer-review-history/1/010f019390479261-e23ca9a1-f283-4691-9bf0-8e7c3ab6e99c-000000/pnhNWmfAE2dACK086mXLvbQCwz0zxKQdnGhWgYdg2yw=187)). If published, this will include your full peer review and any attached files.

If you choose “no”, your identity will remain anonymous but your review may still be made public.

**Do you want your identity to be public for this peer review?** For information about this choice, including consent withdrawal, please see our [Privacy Policy](https://track.editorialmanager.com/CL0/https:%2F%2Fwww.plos.org%2Fprivacy-policy/1/010f019390479261-e23ca9a1-f283-4691-9bf0-8e7c3ab6e99c-000000/Orml0RcduA0ja0n_l8ok9qor6c8qH4A7ot3dwBI7w2A=187).

Reviewer #1: No

Reviewer #3: No

Reviewer #5: No
